# Complete Genome Sequence of Bacillus velezensis Strain DKU_NT_04, Isolated from a Traditional Korean Food Made from Soybeans (Cheonggukjang)

**DOI:** 10.1128/MRA.00477-20

**Published:** 2020-06-11

**Authors:** Man-Seok Bang, Hee-Won Jeong, Yea-Jin Lee, Sang-Cheol Lee, Gi Soo Lee, Sungyong Kim, Han-Hwi Lee, Jang-In Shin, Chung-Hun Oh

**Affiliations:** a Department of Medical Laser, Graduate School, Dankook University, Cheonan, Choongnam, Republic of Korea; b Clinical Trial Institute, Dankook University, Cheonan, Choongnam, Republic of Korea; c Department of Oral Physiology, College of Dentistry, Dankook University, Cheonan, Choongnam, Republic of Korea; University of Rochester School of Medicine and Dentistry

## Abstract

In the present work, we report the complete genome sequence of Bacillus velezensis DKU_NT_04, isolated from cheonggukjang, which is a traditional Korean fermented soybean paste. The final genome assembly consists of a 4.328-Mbp chromosome with 4,134 coding sequences and a G+C content of 45.21%.

## ANNOUNCEMENT

*Bacillus* species are ubiquitous, endospore-forming, Gram-positive bacteria that are of high economic importance due to their specific characteristics, such as their ability to colonize plants; to produce spores, biofilms, and antibiotics; and to induce the synthesis of plant hormones (1). Bacillus velezensis, which is a Gram-positive, rod-shaped bacterium belonging to the class *Bacilli*, has been widely used as a biological control agent in the agricultural field due to its strong ability to suppress plant-pathogenic fungi (2). Bacillus velezensis is frequently isolated from various niches, fermented foods, water, and soil (3, 4). Cheonggukjang is a traditional fermented Korean food that is manufactured by the short-term fermentation of soybeans using *Bacillus* spp., and it contains many proteins, microorganisms, and bioactive compounds (5–7). Here, we report the complete genome sequence of *B. velezensis* strain DKU_NT_04, which is used for high-quality fermented foods.

We purchased 20 cheonggukjang samples in South Korea. To isolate a *B. velezensis* strain, 10 g of cheonggukjang was suspended with 40 ml of sterile water and then heated at 80°C for 30 min. This suspension was serially diluted with sterile water. Cheonggukjang samples were spread onto nutrient agar containing 1% skim milk for 48 h at 37°C. The isolates showing mucolytic ability were selected as the candidates. One strain, DKU_NT_04, which demonstrated the greatest proteolytic ability, was chosen and grown on nutrient agar containing 1% skim milk at 37°C. The total genomic DNA of *B. velezensis* DKU_NT_04 was extracted using the Wizard genomic DNA purification kit (Promega, CA, USA), following the manufacturer’s instructions. The quantity and quality of isolated DNA were determined using a NanoDrop spectrophotometer.

The whole genome of *B. velezensis* DKU_NT_04 was sequenced with a 20-kb SMRTbell library (PacBio DNA/polymerase binding kit P6) on the RS II sequencing platform (Pacific Biosciences, USA) using C4 chemistry with 8 single-molecule real-time (SMRT) cells at Macrogen (Seoul, Republic of Korea) (8). A total of 122,549 PacBio subreads (average subread length, 8,980 bp; *N*_50_, 13,038 bp) were filtered, as per the read qualities. The cleaned reads were then *de novo* assembled using the RS Hierarchical Genome Assembly Process (HGAP) protocol v3.0, short subreads were aligned on long subreads with Basic Local Alignment with Successive Refinement (BLASR), the assembly was performed with Celera Assembler, and the result was polished with error correction by Quiver v1 in SMRT Portal 2.3 (9–11). Default parameters were used for all software unless otherwise specified. This resulted in 3 contigs, which consist of 1 closed circular chromosome of 4,166,265 bp (G+C content, 45.6%; coverage, 192×; GenBank accession number CP026533) containing 2 contigs. The size of one contig is 143,634 bp (G+C content, 35.1%; coverage, 354×; CP026534); the other is 18,288 bp (G+C content, 40.5%; coverage, 957×; CP026535). The genomes were annotated with Prokka v1.12b software (12). There are 4,320 genes, 4,206 coding DNA sequences (CDSs), 27 rRNAs, 86 tRNAs, and 1 transfer-messenger RNA (tmRNA) on the chromosome; 177 genes, 176 CDSs, and 1 tRNA on one contig; and 21 genes and 21 CDSs on the other contig.

### Data availability.

The complete genome sequence of *B. velezensis* strain DKU_NT_04 was deposited in GenBank under the accession numbers CP026533, CP026534, and CP026535. The associated BioProject, BioSample, and SRA accession numbers are PRJNA384709, SAMN08427082, and SRR10344596, respectively.
